# Leading With HEART©: Equine-Assisted Leadership Training Fosters Emotional Intelligence, Resilience, and Identity Formation in First-Year Osteopathic Medical Students

**DOI:** 10.1007/s40670-026-02686-8

**Published:** 2026-03-17

**Authors:** Barb Lutz, Jessica Scheve, Stacey Lilley, Lynn Bohecker, Vasti Holstun, Kim Love, Jon C. McKinnon, Amanda Strivings, Teresa Ramerth, Scott Severance

**Affiliations:** 1 Liberty University College of Osteopathic Medicine, 306 Liberty View Lane, Lynchburg, VA 24502 USA; 2 School of Behavioral Sciences, Liberty University, Lynchburg, VA USA; 3 K. R. Love Quantitative Consulting and Collaboration, Athens, GA USA; 4 Nashville Rescue Mission, Nashville, TN USA; 5 Striving Onward Counseling Services, Leander, TX USA; 6 Psychiatry, Liberty University College of Osteopathic Medicine, Lynchburg, VA USA; 7 Department of Molecular and Cellular Sciences, Liberty University College of Osteopathic Medicine, Lynchburg, VA USA

**Keywords:** Holistic equine-assisted resilience training (HEART©), HeartMath^®^, Well-being, Resilience, Equine-assisted learning, Emotional intelligence, Medical education

## Abstract

**Purpose:**

Burnout, emotional dysregulation, and lack of resilience are prevalent among medical students and professionals contributing to stress and burnout. Despite recognition that emotional regulation, leadership capacity, and PIF are essential competencies, medical education provides limited structured training in these domains. The Holistic Equine-Assisted Resilience Training (HEART©) curriculum was developed to address these gaps through integration of HeartMath^®^ biofeedback, equine-assisted learning and experiential leadership development. This study evaluated the effects of HEART© on physiological regulation, well-being, resilience, and leadership-related competencies among first-year osteopathic medical students.

**Methods:**

A mixed-methods, nonrandomized pretest–posttest design compared a treatment group receiving the five-week HEART© curriculum intervention with a control group that did not receive training. Quantitative measures included heart rate variability for those in the treatment group, the Medical Student Well-Being Index, and the 14-item Resilience Scale completed by both the treatment and control group. Qualitative data were collected via semi-structured interviews of the treatment group participants and analyzed using inductive phenomenological thematic analysis.

Qualitative analysis identified themes of increased emotional awareness, improved selfregulation, and greater alignment between thoughts (head) and feelings (heart). Participants described horses as providing immediate, nonjudgmental feedback that facilitated experiential learning.

**Results:**

Treatment group participants demonstrated significant improvements in heart rate variability and coherence and MSWBI scores, indicating improved autonomic regulation and reduced stress. The analysis reflected a significant increase in resilience for the treatment group compared with the control group.

**Conclusions:**

The HEART© curriculum was associated with measurable physiological and psychological benefits and supported development of emotional regulation and leadership-related competencies in OMS-1 participants.

## INTRODUCTION

Healthcare professionals face many challenges. Personal sacrifices of time and finances, combined with increased corporate oversight, escalating workloads, and rising societal expectations have intensified burnout across the health professions that is recognized as a major contributing factor in the global health professionals shortage [1]. Current projections estimate a global shortfall of ~ 11 million healthcare providers by 2030, a deficit that will leave billions without adequate access to essential services [2, 3]. Alarmingly, burnout is no longer confined to practicing clinicians; systematic reviews indicate that many future physicians begin experiencing symptoms before residency, underscoring the need for preventive interventions within medical education [4, 5].

Burnout is characterized by emotional exhaustion, depersonalization, and diminished personal accomplishment, components deeply intertwined with the emotional demands of healthcare work [6]. Medical professionals frequently experience powerful, rapidly changing emotions including fear, shame, grief, uncertainty and hope, all in a day’s work. When these emotions are suppressed rather than processed, they can manifest as anxiety, depression, or impostor phenomenon, impairing mental health and professional development [7, 8]. Because professional identity formation (PIF), ethical decision-making, and resilience have deeply personal and emotional roots, students often mask distress behind a façade of competence, and reinforce maladaptive coping strategies that persist into residency and practice. Notably, hope, a key driver of motivation and learning, has been associated with lower stress and burnout among medical students and may mitigate suicide risk, given well-documented links between distress and suicidal ideation in trainee populations [9, 10].

Emotions exert powerful physiological effects. Emotional dysregulation disrupts autonomic balance and contributes to cognitive overload; by contrast, effective self-regulation supports adaptability and sustained engagement [11–13]. Resilience, defined as the capacity to adapt to stress and recover from adversity, mitigates burnout risk and strengthens performance. Importantly, resilience is not fixed; meta-analyses demonstrate it can be cultivated through structured training [14, 15]. HeartMath^®^ coherence practices represent one such approach to improving emotional regulation, HRV, and stress recovery via evidence-based techniques and real-time biofeedback; randomized data in coronary disease show HRV biofeedback improves myocardial perfusion responses to mental stress, illustrating physiologic impact [16, 17].

Emotional intelligence (EQ) is similarly essential to effective medical practice. Higher EQ predicts stronger academic performance, improved patient satisfaction, enhanced teamwork, and more effective clinical decision-making [18–20]. Traditional curricula favor biomedical knowledge and technical skills over emotional, relational, and reflective competencies needed for sustainable professional growth.

Leadership development represents another gap in undergraduate medical education (UME). Reviews and commentaries describe highly variable, often elective leadership offerings, with no widely adopted, standardized framework in U.S. UME [21–24].

PIF, commonly described as “thinking, acting, and feeling like a physician,” has long been recognized as a central goal of medical education. The Carnegie 2010 report explicitly called for curricula that “cultivate the formation of professional identity,” yet many programs still privilege scientific knowledge over the personal, emotional, and relational competencies required for sustained professional development [25, 26]. Although the Association of American Medical Colleges’ 2023 competency refresh articulates expectations for incoming students (e.g., cultural humility, resilience/adaptability, teamwork and collaboration, and commitment to learning and growth) [27], much of the literature continues to emphasize science skills rather than the developmental processes that underlie professional growth. Consequently, students often equate their worth with achievement and the emotional component associated with burnout, impostor syndrome and distress is overlooked [5, 7, 8].

To address these challenges, the first author developed the Holistic Equine-Assisted Resilience Training (HEART©) curriculum which is an experiential program that focuses on strengthening physiological, psychological, emotional, spiritual, and interpersonal connections to help participants build resilience, increase well-being, develop leadership skills, establish emotional balance, and reduce the risk of burnout. HEART© integrates HeartMath^®^ biofeedback, structured leadership development, and equine-assisted learning (EAL). EAL is an experiential modality in which horses provide immediate, nonjudgmental feedback mirroring human relational dynamics. Horses’ innate sensitivity to incongruence (fear, uncertainty, self-doubt) offers real-time insight into internal processes and interpersonal effectiveness, supporting competencies aligned with the ACGME core competencies often underdeveloped in trainees [28].

## Study Rationale

Instruction in emotional regulation, PIF, and leadership remains limited in the context of format medical education. The HEART© curriculum was designed to address this gap by combining biofeedback, experiential learning, and relational leadership training. Prior EAL literature also links equine-based interventions with improved well-being and strengthened leadership competencies among students in the health professions [29–31]. We set out to evaluate the effectiveness of the HEART© curriculum to increase well-being, resilience, emotional intelligence, and leadership competencies in first-year osteopathic medical students (OMS-I) (Table 1).

**Table 1:** HEART© curriculum synopsis

*Session Title*	*Key Concepts*	*ACGME Core Competencies*
Physiological Connections	“GRACE” pose HeartMath^®^^*^ Quick Coherence Technique	Interpersonal & Communication Skills
Psychological Connections	Identifying Limiting Beliefs Conquering Fear and Self-doubt Overcoming Obstacles	Practice-Based Learning
Emotional Connections	Boundaries Appreciation and Gratitude Forgiveness	Self-Care / Well-Being
Spiritual Connections	The Power of Intention Passion and Purpose Mission and Vision	Interpersonal & Communication Skills
Interpersonal Connections	Mapping and Direction Setting Decision Making with Confidence Leadership Competency	Professionalism

HeartMath® involves the mathematical analysis of heart rate variability, a marker of physiological coherence in healthy states that becomes disrupted when an individual experiences stress or illness. HeartMath® and HEART© are different concepts. HEART© is the acronym for the leadership program tested in this study.

## METHODS

### Study Design

This study employed a mixed-methods design to evaluate the effect of the HEART© curriculum on physiological regulation, well-being, resilience, and leadership skills among first-year osteopathic medical students (OMS-I). Quantitative and qualitative data were collected immediately before and after the five-week training, analyzed independently, and integrated during interpretation to provide a comprehensive understanding of participants’ experiences. The quantitative component used a nonrandomized pretest–posttest design with a treatment group receiving the HEART© intervention and a control group receiving no training. The qualitative component used a phenomenological approach to explore participants’ lived experiences of the curriculum.

### Participants and Recruitment

In spring 2022, all 154 OMS-I students at the institution received an email invitation describing the study and the voluntary nature of participation. The email explained that the HEART© curriculum integrated HeartMath^®^ biofeedback, EAL, and leadership development, and that participants would complete pre- and post-intervention assessments of HRV, well-being, and resilience. Students were informed that interviews would be conducted by a third party following the program. Students were informed that they would receive a gift card for participating in the control group.

Students who could commit to attending all five weekly in-person sessions were assigned to the treatment group. Those unable to remain in the area for the duration were placed in the control group and completed only the pre- and post-intervention surveys. Initially, 21 students enrolled (10 treatment, 11 control). One treatment group participant withdrew, and the data from two control group participants was removed due to straight-lining on surveys. The final sample included 18 participants (9 treatment, 9 control). Demographic characteristics are summarized in Table 2.

**Table 2:** HEART© Participant Demographics

Participants	Treatment	%	Control	%
Total Number	9	100	9	100
Race				
White	7	78	7	78
Asian	1	11	2	22
Prefer Not to Answer	1	11	0	0
Gender				
Female	9	100	4	44
Male	0	0	5	56
Marital Status				
Single	8	89	6	67
Married	1	11	3	33
Prior Horse Experience	1	11	N/A*	

*N/A Not Applicable

### HEART© Curriculum Intervention

The HEART© curriculum was developed by the principal investigator, who is a certified Equine-Assisted Coaching Association instructor and certified HeartMath^®^ mentor. HeartMath^®^ involves the mathematical analysis of heart rate variability, a marker of physiological coherence in healthy states that becomes disrupted when an individual experiences stress or illness. HeartMath^®^ and HEART© are different concepts. HEART© is the acronym for the leadership program tested in this study. The five-week program consisted of weekly 2.5-hour sessions combining classroom-based instruction with equine-assisted experiential exercises. Each session introduced concepts related to physiological regulation, emotional intelligence, resilience, leadership, and self-awareness, followed by EAL activities designed to solidify these concepts.

Participants in the treatment group attended HEART© curriculum sessions once a week for 2.5 h for five consecutive weeks. Each session included a didactic lesson held in a classroom setting followed by an EAL session conducted in a fenced corral. During the EAL session, participants were instructed to invite one or more horses to partner with them in completing a task, without physically controlling the horses. The horses were free to choose whether to engage with the participant. No additional instructions were provided, allowing participants to apply the classroom concepts in their own unique manner. Voluntary engagement by the horse signified the participant’s ability to establish coherence and build connections. The program director and an equine specialist supervised all equine-assisted learning activities. Life coaching techniques were integrated throughout the session to facilitate reflection, self-awareness, and meaning-making. Participants were encouraged to maintain reflective journals and completed semi-structured interviews within two weeks of completing the program.

### Heart Rate Variability (HRV) Measurement

HRV was assessed using HeartMath^®^ emWave^®^ Pro Plus software and an infrared pulse plethysmograph ear sensor. Upon arrival on the first and last days of the intervention, participants sat quietly in a rocking chair for five minutes before recording began. HRV was recorded for three minutes following the HeartMath^®^ three-step protocol. Higher HRV and coherence values indicate greater autonomic balance and emotional regulation capacity.

### Medical Student Well-Being Index (MSWBI)

The MSWBI is a validated seven-item instrument assessing fatigue, depression, burnout, anxiety, stress, and quality of life. Each “Yes” response receives one point, yielding a total score from 0 to 7. Higher scores indicate greater levels of stress and lower well-being. All subjects completed the MSWBI pre- and post-intervention via Qualtrics.

### Resilience Scale (RS14™)

The RS14™ survey is a validated 14-item measure of psychological resilience rated on a 7-point Likert scale. Items assess five subdomains: Self-Reliance, Purpose, Equanimity, Perseverance, and Authenticity. Total scores range from 14 to 98, with higher scores indicating greater resilience. All subjects completed the online RS14™ questionnaire pre- and post-intervention using the Qualtrics platform with the site license provided by Liberty University.

### Qualitative Methodology

A phenomenological approach was used to explore participants’ lived experiences of the HEART© curriculum. Semi-structured interviews were conducted by trained third-party interviewers to minimize bias. Interviews were audio-recorded, transcribed verbatim, and reviewed by the qualitative analysis team. Because no established theoretical framework exists for EAL within medical education, an inductive analytic approach was used. Open, line-by-line coding captured participants’ explicit statements, which were then compared across transcripts and consolidated into descriptive categories representing recurring patterns. This approach ensured that themes remained grounded in participants’ narratives.

### Coding Procedures and Trustworthiness

Trained qualitative analysts independently coded each transcript. A shared codebook was developed collaboratively and refined through iterative discussion. Codes were organized into themes, which were reviewed by the qualitative methodologist and doctoral-level researchers trained in phenomenological methods. An audit trail, analytic memos, and reflexive dialogue enhanced dependability and confirmability. Themes were member-checked with participants to ensure accuracy and resonance.

### Statistical Analysis

We analyzed the HRV data using IBM SPSS 29 software with *p* = .05. Assumptions for *t*-tests were independence of observations, no significant outliers in the groups, normality, and homogeneity of variance. The independence of observations was established through the design of this study. There were no outliers in the groups, and each group appeared to show normality and homogeneity of variance in preliminary testing of the data with Levene’s test for equality of variances.

To account for the repeated measures within participants when comparing MSWBI and RS-14 outcomes across the control and treatment group, we used a linear mixed effects model with an interaction of group and pre/post for both MSWBI and RS-14™, while comparing the outcomes of the two groups. This model requires that the groups be independent of one another, which was established through the design of the study. However, the model includes a random intercept, which accounts for the lack of independence of paired pre- and post-intervention observations from each individual. Both analyses included pre-planned comparisons of pre- and post-intervention scores within each group of participants, regardless of the significance of the test of the interaction comparing the two groups. A level of significance of 0.05 was used for all statistical analyses. Model residuals were checked for both outcomes, and approximate normality within each combination of group and timepoint was determined using Shapiro-Wilk tests, as well as visualizations using histograms and normal QQ plots. Skew and kurtosis values of the individual treatment groups at both timepoints were also used to check normality and found to be within acceptable limits [32].

Levene’s test determined that there was no evidence of heterogeneity across the groups and timepoints within each model. No outliers were observed.

## RESULTS

### Physiological Outcomes

The HEART© curriculum yielded measurable improvements in participants’ physiological regulation. Prior to the intervention, HRV recordings from treatment-group participants exhibited the jagged, irregular patterns characteristic of an incoherent autonomic state. These patterns are commonly associated with heightened stress, emotional dysregulation, and reduced physiological resilience. After the five-week training, recordings were smoother, and more sinusoidal, which reflect the increased coherence and improved autonomic balance in the trainees. One participant described this shift succinctly: *“Most valuable was learning to calm myself. My grounding calmed the horse too.”*

**Fig. 1:**
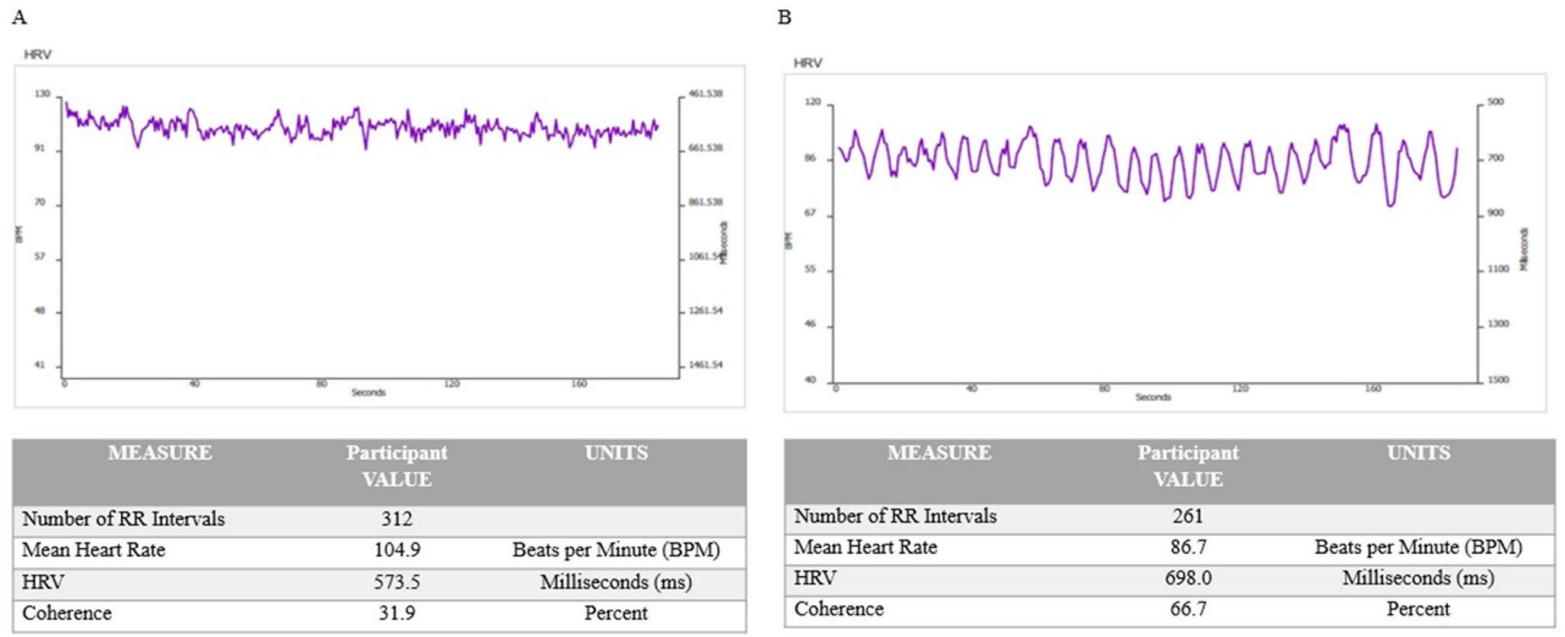
HEART© intervention increased HRV, as evidenced by participant recordings. **A **HRV recording and metrics from participant 7 before HEART© intervention. Data was collected during a single, three-minute recording using an infrared pulse plethysmograph ear sensor attached to the participant’s ear lobe and analyzed using HeartMath emWave pro software. The y-axis on the left reports beats per minute (BPM), while the y-axis on the right reports HRV in milliseconds. The HRV values are reported in descending fashion, i.e., higher values are closer to the x-axis. (Although they are reported in the table, coherence and number of intervals are not shown on graph for clarity). **B** HRV recording and metrics from participant 7 after five weeks of HEART© intervention

For the representative participant shown in Fig. 1, coherence increased 35% from pre- to post-intervention.

Similar changes were observed at the group level. Treatment-group participants demonstrated statistically significant increases in HRV, and coherence (*p* ≤ .05).

Participants often described applying these physiological skills in everyday situations. One student noted, *“I found myself going straight into coherence automatically during stressful moments.”* Complementing these findings, heart rate decreased slightly while HRV increased, which suggested more stable and organized cardiac rhythms in trainees.

**Fig. 2:**
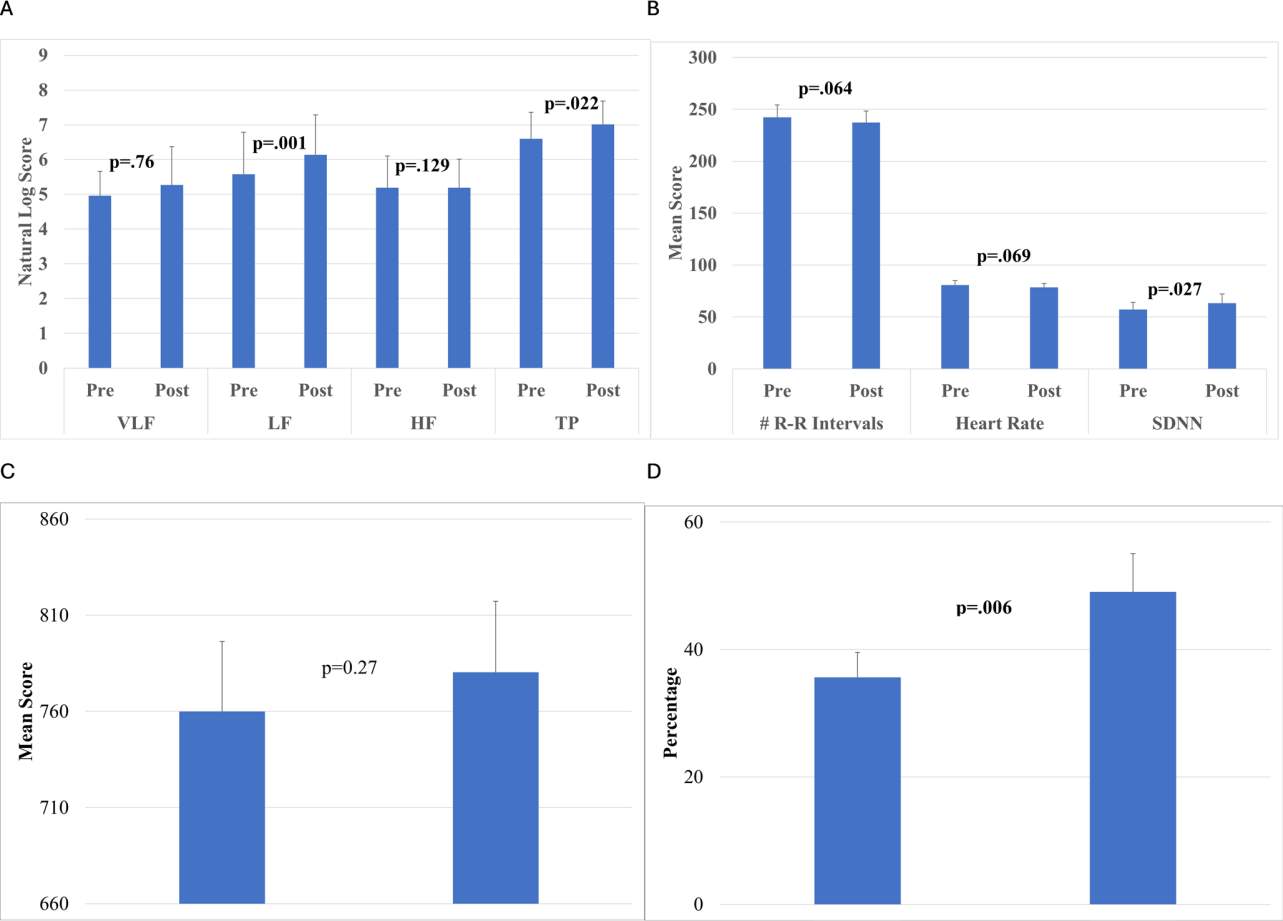
HEART© increased HRV and coherence. For each of the panels of Fig. 2 standard error values were calculated by dividing the standard deviation by the sample size square root. **A** Recordings of very low frequency (VLF), low frequency (LF), high frequency (HF) and total power (TP). To normalize the parameters across treatment group participants, values were transformed to their natural logarithmic values. **B** Number of R-R intervals, heart rate, and standard deviation of the number of all normal intervals (SDNN) for treatment group participants. The number of R-R intervals represents the average time in milliseconds between two successive R waves. Heart rate (HR) in beats per minute is the average heart rate of treatment group participants. **C** HRV, defined as the time in milliseconds between R-R intervals, increased as a result of the treatment intervention. **D** Coherence, expressed as a percentage with values ranging from 0-100%, increased following HEART© intervention. As with HRV, higher values are desirable

Collectively, these quantitative and qualitative outcomes indicate that participants not only learned coherence-building techniques but were able to shift their physiological state toward coherence (Fig. 2).

### Well-being and Resilience

Table 3 includes summary statistics, describing pre- and post-implementation RS-14™ and MSWBI scores for both control and treatment groups. The results of the linear mixed effects model for the MSWBI showed that the MSWBI outcome for the treatment group is the only statistically significant change at the 0.05 level of significance (t (16) = -2.22, *p* = .041), where the mean decreased from 4.22 (SE = 0.601) to 3.00 (SE = 0.601). There was no evidence of a difference between the two groups as a result of the treatment as measured by the model interaction term (F (1,16) = 1.31, *p* = .270).

Qualitative feedback supported these quantitative findings. Participants commonly described renewed emotional capacity and a greater ability to pause, reset, and engage more intentionally in their daily lives. One participant reflected, *“Taking real mental breaks and being present brought back joy.”* This narrative aligned with participants’ reports of improved coping, increased resilience, and a greater sense of balance following the intervention.

From the linear mixed effects model for the RS-14™ survey, individually, neither group shows a statistically significant change for the RS-14™ survey; interestingly, a statistically significant difference occurred between the changes in the two groups as measured by the model interaction term (F (1,16) = 4.94, *p* = .041). The control group experienced a downward trend while the treatment participant group experienced an upward trend in resilience scores.

Participant feedback aligned with these quantitative patterns. Students frequently described feeling better able to recover from stressors and maintain emotional steadiness across academic and clinical demands. One participant expressed this shift succinctly: *“This helped me avoid the burnout slide—regulate*,* refocus*,* and move on.”*

**Table 3:** Linear mixed effects analysis of MSWBI and RS-14™ survey responses of control and treatment group participants

Variable	Group	Time	Mean	Std. Dev.	Median	Minimum	Maximum	Skew	Kurtosis
RS-14									
	Control	Pre	83.0	7.37	86	68	92	–1.151	1.146
		Post	80.9	9.69	84	61	91	–1.041	0.939
	Treatment	Pre	84.9	4.54	86	76	90	–1.000	0.416
		Post	87.9	5.40	90	79	94	–0.603	–1.078
MSWBI									
	Control	Pre	4.22	1.39	4	2	6	–0.146	–1.060
		Post	3.89	2.15	5	1	7	–0.011	–1.725
	Treatment	Pre	4.22	1.48	5	1	6	–1.375	2.186
		Post	3.00	2.06	3	0	5	–0.220	–1.932

Summary statistics for the RS-14™ and MSWBI scores comparing 9 control and 9 treatment student volunteer subjects before and after the HEART© curriculum intervention in a non-randomized pretest-posttest design. Skew and kurtosis values are within common rule-of-thumb limits indicating the assumption of normality is not violated.

### Qualitative Findings

As coding progressed, distinct themes began to become apparent in answers from all participants. Once all transcripts were analyzed and themes finalized, it became clear that the findings could be cohesively and concisely organized using the acronym **BALANCE**. As one participant observed, *“Balance. That’s what health is… When there’s an imbalance*,* you have disease.”* During member checking, treatment group participants affirmed that the acronym BALANCE accurately represented their experiences.

#### B-Belonging

Positive and meaningful interactions are essential to fostering a sense of belonging. Imposter syndrome, marked by self-doubt and fear of being exposed as a fraud, can undermine that sense of belonging and contribute to anxiety and depression. Treatment group participants reported that the EAL experience strengthened their understanding of how emotional self-awareness and compassion support connection. Several students described how the HEART© curriculum equipped them with coping strategies that enabled clearer thinking, more confident decision-making, and reduced self-doubt. As one participant explained, *“I didn’t feel like I had to pretend. Everyone there was trying to figure things out*,* just like me.”* Another added, *“For the first time in medical school*,* I felt like I was part of a group where we were allowed to be human.”*

Participants highlighted the powerful role that equine interactions played in fostering a sense of belonging and self-connection. Moments of attunement with the horses invited reflection on trust, boundaries, and vulnerability, and many participants noted that the horses’ immediate, unfiltered responses often surfaced emotions they had not yet recognized in themselves. One participant reflected, “*I think this was a really cool thing because it’s helping me find my authentic self. It’s helping me listen to myself more and be more in tune with my emotions*,* which I think would help so much with burnout*.” Another described a moment of personal clarity: “*Through this experience I am becoming more aware of what makes me*,* me; and I like who I am. I am finding a confidence that I did not know I had*.” Collectively, these experiences underscored how emotional clarity and congruence support authentic connection—both with the horses and with other people.

#### A-Awareness

Participants in the treatment group explained that their prior understanding of emotions and coping strategies left them uncomfortable with their own feelings, leading them to suppress emotions or label them as “good” or “bad.” Through the HEART© curriculum and EAL activities, they learned to tolerate and interpret their emotions rather than avoid them. This shift replaced previous tendencies to please others or conform to external expectations with healthier emotional expression. Another participant stated, “…*before this program*,* I didn’t even realize I was burying my emotions and not talking nice of myself mentally.”* As participants became more accepting of what they were thinking and feeling, they reported greater clarity and improved ability to engage constructively with others’ emotions. As one participant reflected, *Thinking that this is what I really want and then having visual evidence in front of your eye that it’s not what you’re communicating at the very least is just a profound visualization of all the things that you’re working through on the inside*.”

#### L-Lens

Treatment group participants consistently noted that the unspoken norms of medical training promoted suppressing emotions, being skeptical, and prioritizing performance over mental or emotional well-being. Many felt that meeting external standards, regardless of their internal emotional state, was implicitly valued. This contributed to the belief that emotions such as empathy were signs of weakness and could hinder academic success, professional advancement, and critical thinking. A participant noted, *“I’m truly amazed at how much I’m leaning in this research study each week. It’s really helped to start changing my mindset for the better.”*

Before the HEART© curriculum, participants described how their difficulty understanding and tolerating their own emotions made it challenging to empathize with themselves or others.

#### A-Acceptance

Participants shared that the HEART© curriculum empowered them to more fully tolerate and accept their emotions, shifting from avoidance to constructive engagement. Several described how developing this acceptance made emotionally complex interactions less overwhelming. One participant explained, *“I could tell when I’d switched out of calm and bring myself back*,*”* illustrating new confidence in recognizing and regulating emotional shifts. After learning to identify and understand their feelings, they reported becoming more comfortable engaging with others’ emotions as well. Another participant noted, *“When the horse finally approached me*,* it felt like it really ‘saw’ me. Like it could tell I was being honest with myself*,*”* reflecting how coherence deepened their sense of relational grounding.

This increased emotional acceptance also reshaped their understanding of professionalism. Learners described recognizing that emotional presence was not incompatible with competence and one participant summarized, *“I realized emotions aren’t the enemy. They’re information.”* Through HEART©, participants discovered that a physician can be both decisive and compassionate simultaneously and that embracing, not suppressing their internal states enabled more effective and authentic interpersonal engagement.

#### N-New Coping

Participants reported an increased ability to engage right-brain processes such as creativity, intuition, and imagination during EAL sessions. As their learning progressed, many described developing a more effective balance between analytic and intuitive thinking, which they associated with greater emotional well-being and cognitive flexibility. Several explained that the HEART© coping strategies helped them remain centered in challenging situations, which enabled them to acknowledge and manage emotions without becoming overwhelmed. One participant noted, “*it was a very insightful day for me because I’ve got to break past that habit of internalizing things*,* and that’s usually what then would lead to fermenting on emotions and causing physiological distress.”*

Participants also described becoming more attuned to their internal states and more skilled in regulating them. Many recognized longstanding patterns of emotional suppression and reported developing healthier approaches to managing stress, fear, and self-doubt; skills they viewed as essential to their future clinical roles. Participants mphasized that HeartMath^®^ coherence techniques provided practical, accessible tools for calming the mind and body during moments of heightened stress, and for enhancing their capacity for self-regulation in both educational and potential clinical environments. One trainee reflected, “*It’s crazy how well the exercises work at clearing my mind and making decisions easier*,” demonstrating increased confidence in their emotional regulation skills.

#### C-Confidence

Participants described gaining greater emotional awareness and an increased ability to process their emotions constructively. They felt these changes directly strengthened their confidence and capacity for compassion. Many explained that learning to reflect on, rather than suppress, their emotions empowered them to respond more empathetically and address challenges more directly. One student noted, *“During the exercises I felt more confident in myself. I was living in the moment and was relaxed.”* A participant who was concurrently shadowing a physician in a free clinic shared, “*I think it helped a lot with having a better presence with patients and how you influence them. Being more aware of my thoughts and then my actions and my nonverbal cues I think will be very important not just with patients but in life in general.”* illustrating a shift toward viewing emotions as a resource in the clinic rather than a liability.

Participants also recognized that confidence plays a critical role in patient trust and professional identity. Several acknowledged that self-doubt had previously limited their ability to engage authentically with patients and peers. After completing the HEART© curriculum, students reported increased self-assurance and a stronger ability to focus on others. As one participant shared, *“I just think the confidence building*,* the leadership*,* the goal setting is all going to be very positive for when I do become a doctor and when I have my own patients*.” reflecting a move toward greater confidence in clinical interactions.

Equine activities further illuminated discrepancies between intention and action, prompting deeper reflection on PIF. Students described how the horses’ responses often revealed their internal emotional states. One participant stated, *“The horse showed me when I wasn’t as grounded as I thought*,*”* which encouraged them to cultivate greater alignment between their internal state (heart) and outward behavior. Collectively, participants reported feeling more grounded, confident, and connected to their developing sense of purpose in medicine.

#### E-Experiential Learning

Treatment group participants explained that the EAL sessions helped them better understand the power of emotions, the importance of coping skills, and the ways in which their interactions with horses mirrored their interactions with people. Working with the horses required them to confront their own emotions and intentions; as one student noted, *“The horse mirrored what I was feeling. When it wouldn’t come near me*,* I realized I wasn’t as calm or open as I thought.”* Learners described how horses either approached or avoided them depending on their emotional state, making the effects of emotional regulation immediately visible. Another participant shared, “*If your heart doesn’t match our head*,* the horse will call you out on your B.S. ha ha!”*

Participants recognized that their emotional energy influenced not only the horses but also the people they interacted with. Many drew parallels between equine behavior and patient behavior, acknowledging that, like horses, patients may not automatically cooperate and respond to a clinician’s presence, coherence, and clarity. Participants consistently described the horses as responsive, nonjudgmental partners whose behavior provided real-time feedback on leadership presence. They learned that horses responded not to verbal commands but to emotional congruence, intentionality, and nonverbal communication. As one participant shared, “*The horses seem like they can sense everything. When you get in with the horses*,* if you don’t actually believe something you’re saying*,* they know. And it makes it so much more real.”*

These experiences strengthened participants’ confidence in their ability to influence others through a calm, focused presence and deepened their understanding of relational leadership. One student reflected, “*I was used to being told exactly what to do*,* and this helped me think for myself*.” Participants noted that the integration of didactic instruction, biofeedback, and equine interaction made concepts such as resilience, emotional regulation, and leadership more concrete and applicable to clinical training. Many described these experiential components as more impactful than traditional classroom instruction. As one student explained, “*You can change a lot more when you cut through everything*,* and that’s what the horses let you do.*”

### Mixed Methods Integration

The integration of quantitative and qualitative yielded similar results. Physiological improvements in HRV and coherence aligned closely with participants’ descriptions of enhanced emotional regulation and greater calm. Similarly, increases in MSWBI scores were consistent with qualitative reports of reduced stress and improved well-being. Divergence in resilience trajectories between groups was echoed in participants’ narratives, as treatment group members described developing new coping strategies and a clearer sense of purpose. One student explained, *“Practicing when I didn’t need it made coherence automatic when I did*,*”* illustrating how regulation skills became accessible during high-stress moments. Taken together, these findings suggest that the HEART© curriculum supported both the physiological and psychological dimensions of resilience, and offered a holistic approach to well-being and leadership development in early medical education.

## Discussion

This mixed-methods study examined the utility of the HEART© curriculum, a multisensory program integrating HeartMath^®^ biofeedback, EAL, and experiential leadership development, on physiological regulation, well-being, resilience, and leadership-related competencies among first-year osteopathic medical students. The quantitative findings demonstrated that the curriculum produced meaningful improvements in HRV, coherence, resilience and well-being, while qualitative data revealed transformative shifts in emotional awareness, leadership presence, and PIF. Together, these results suggest that the HEART© curriculum may help address the emotional, relational, and developmental challenges common in healthcare.

Although this initial cohort included only female students, the findings remain relevant given that women currently constitute the majority of U.S. medical school matriculants (55.1% in 2024–2025) and the majority of total MD enrollment [5, 32]; in academic medicine, women now represent ~ 45% of full-time faculty overall, with gains in several leadership roles, a context consistent with a female-only sample but still underscoring the need for gender-diverse cohorts in future work [33].

### Physiological Regulation and Emotional Regulation

The significant improvements in HRV and coherence in the treatment group indicate enhanced autonomic regulation and increased capacity for emotional regulation. HRV is widely recognized as a peripheral index of stress resilience, executive functioning, and adaptive emotion regulation within the neurovisceral integration framework [11–13]. The observed shifts with once-weekly HeartMath^®^ practice are consistent with randomized data showing that HRV biofeedback can improve physiologic responses to mental stress [16] and with large-scale coherence data describing stable resonance frequencies and emotion-linked coherence patterns [17]. The rapid physiological changes observed underscore the potential of biofeedback-supported training to provide students with practical strategies for managing the emotional demands of medical school.

### Well-Being and Burnout Prevention

The significant improvement in MSWBI scores among HEART© participants suggests increased ability to handle stressors and enhanced well-being post-intervention, a notable departure from typical preclinical trajectories characterized by rising distress [5, 34]. The lack of improvement in controls and a downward trend in resilience mirror common early-training patterns. Qualitative reflections of feeling calmer, more grounded, and better able to manage stress reinforce the quantitative findings and highlight the value of integrating well-being practices early in medical school training.

### Resilience

Quantitative data indicated that the HEART© curriculum stabilized or enhanced resilience (RS-14), even with this small sample and without a statistically significant within-group RS-14 change. Prior systematic reviews also show resilience is trainable, [14, 15] supporting the potential of integrated physiological-cognitive-emotional programs. Students’ narratives (new coping strategies, reframing challenges, authenticity) align with RS-14 conceptual domains and validation work [35]. Resilience-building interventions may be most effective when they combine cognitive, emotional, and physiological components, which may have facilitated deeper integration of resilience-related skills than traditional didactic instruction alone.

### Leadership Development and Professional Identity Formation

EAL uniquely fostered coherence and PIF. Participants described horses as responsive, nonjudgmental partners whose behavior provided immediate feedback on coherence and interpersonal effectiveness, which requires regulation, clarity of intention, and confident, authentic nonverbal communication. These experiences reflect core leadership competencies relevant to clinical teamwork and patient care, [21–24, 31] and echo Carnegie’s call to explicitly cultivate PIF as an educational objective [25, 26].

### EAL as a Catalyst for Transformation

Qualitative findings emphasized that didactics + biofeedback + equine interaction created a potent learning environment that facilitated insight, reflection, and behavioral change. This aligns with experiential learning theory, which emphasizes concrete experience → reflective observation → abstract conceptualization → active experimentation; EAL solidifies this cycle with real-time, experiential feedback that is difficult to reproduce in classrooms [36]. The nature of EAL allowed students to experience concepts such as coherence, intention, and relational presence in real time, making learning more memorable and powerful.

### Mixed-Methods Integration

The agreement between quantitative and qualitative findings strengthens the validity of the results and provides a more comprehensive understanding of how students learned and applied new skills. Increased HRV and coherence corresponded with participants’ descriptions of enhanced emotional regulation and calmness, while improvements in well-being aligned with qualitative reports of reduced stress and increased clarity. The divergence in resilience trajectories between groups was reflected in participants’ narratives of developing new coping strategies and a stronger sense of purpose.

Together, these findings suggest that the HEART© curriculum supports both the physiological and psychological dimensions of resilience, offering a holistic approach to well-being and leadership development in early in a student’s medical education.

### Relevance to Medical Education

Early training in emotional regulation is essential. HRV/coherence improvements show students can learn to regulate physiological and emotional states even with brief weekly training, echoing randomized biofeedback data [16]. Experiential learning enhances leadership development. EAL offers powerful opportunities to develop coherence and interpersonal effectiveness aligned with teamwork and patient care [21–24, 29–31]. PIF requires more than didactics. EAL helps students integrate authenticity, purpose, and self-awareness [25, 26, 36] and supports integration of skills. Hands-on experiences strengthen and integrate emotional and leadership capacities [31, 36]. Well-being interventions should be proactive. Early, skills-based programs may reduce downstream burnout [5, 34]. Partnerships can expand EAL access. Collaborations with community equine centers and certified EAL practitioners can make programming feasible at schools without on-site facilities [31, 33]. Mixed-methods evaluation strengthens curriculum design. Combining physiological, psychological, and qualitative metrics yields a comprehensive view of learning and change.

### Cost Analysis

When a medical student withdraws, the institution loses both the training resources already invested and the future net-tuition revenue the student would have generated, along with the long-term loss of a future physician in a workforce already facing shortages [37–39]. Using round, conservative numbers: if the combined loss of tuition, fees, and prior instructional investment totals about $100,000 per student, and the full cost to deliver the HEART© program for an entire class is about $140,000, then preventing even one student from leaving recovers roughly $100,000 in otherwise lost value. In practical terms, retaining just one student offsets approximately 70% of the full program cost, and retaining two students more than pays for the entire program, illustrating why even small improvements in retention produce a strong positive return on investment.

### Limitations

Several limitations should be considered when interpreting these findings. The sample size was small, with only 18 participants included in the final analysis. Although the mixed-methods design provided rich data, the limited number of participants reduces statistical power and constrains generalizability. Participants were all from the same institution located in the southern U.S. Larger and more diverse cohorts are needed to confirm these results and explore subgroup differences.

This study used a nonrandomized design. Participants self-selected into the treatment or control group based on their availability for in-person sessions, which may have introduced selection bias. Students who chose the HEART© curriculum may have been more motivated for personal development or more receptive to experiential learning. Future randomized controlled trials would strengthen causal inferences.

Gender distribution differed between groups; the treatment group consisted entirely of women. While this reflects voluntary enrollment patterns, it limits the ability to examine gender-related differences in response to the curriculum. Studies with more balanced sampling are warranted.

The intervention required specialized resources, including trained equine specialists, certified HeartMath^®^ instructors, and access to equine facilities. These requirements may limit scalability for institutions without established partnerships or infrastructure. Although the curriculum could be adapted, key elements of equine-assisted learning may be difficult to replicate without horses.

The study relied on short-term pre- and post-intervention assessments. While immediate improvements in HRV, well-being, and qualitative outcomes are promising, the durability of these effects is unknown. Longitudinal follow-up would help determine whether skills acquired through HEART© persist into the clinical years.

The RS-14 survey was entered into Qualtrics backwards but did not affect the results.

Finally, qualitative data were collected only from treatment group participants, which may have introduced positive bias. Although member checking enhanced trustworthiness, including perspectives from nonparticipants or employing additional qualitative methods could provide a more balanced understanding.

Despite these limitations, the study offers valuable insights into the potential of the HEART© curriculum to support emotional regulation, resilience, and leadership development in early medical education.

## Conclusion

The HEART© curriculum demonstrated promising benefits for first-year osteopathic medical students across physiological, psychological, and developmental domains. Improvements in HRV, coherence, and well-being, together with qualitative evidence of enhanced emotional awareness, leadership presence, relational attunement, and emerging professional identity, suggest that experiential approaches integrating biofeedback, emotional-regulation training, and equine-assisted learning can strengthen competencies often underdeveloped early in medical training.

These results illustrate how relational, experiential learning can enrich traditional biomedical training by supporting student well-being and addressing enduring challenges such as burnout, emotional dysregulation, and declining resilience. Although the small sample and nonrandomized design limit generalizability, similar qualitative and quantitative findings provides compelling preliminary support for this integrative educational model.

As medical education continues to evolve, embedding structured emotional-regulation training, EAL and reflective practice may help cultivate more resilient, self-aware, and relationally effective physicians. The HEART© curriculum offers a feasible and innovative model for supporting holistic student development and strengthening the emotional and interpersonal capacities essential for contemporary clinical practice.
